# Vibrotactile stimulation: A non-pharmacological intervention for opioid-exposed newborns

**DOI:** 10.1371/journal.pone.0175981

**Published:** 2017-04-20

**Authors:** Ian Zuzarte, Premananda Indic, Bruce Barton, David Paydarfar, Francis Bednarek, Elisabeth Bloch-Salisbury

**Affiliations:** 1 Department of Neurology, University of Massachusetts Medical School, Worcester, Massachusetts, United States of America; 2 Department of Quantitative Health Sciences, University of Massachusetts Medical School, Worcester, Massachusetts, United States of America; 3 Wyss Institute for Biologically Inspired Engineering, Harvard University, Boston, Massachusetts, United States of America; 4 Department of Pediatrics, University of Massachusetts Medical School, Worcester, Massachusetts, United States of America; TNO, NETHERLANDS

## Abstract

**Objective:**

To examine the therapeutic potential of stochastic vibrotactile stimulation (SVS) as a complementary non-pharmacological intervention for withdrawal in opioid-exposed newborns.

**Study design:**

A prospective, within-subjects single-center study was conducted in 26 opioid-exposed newborns (>37 weeks; 16 male) hospitalized since birth and treated pharmacologically for Neonatal Abstinence Syndrome. A specially-constructed mattress delivered low-level SVS (30-60Hz, 10–12μm RMS), alternated in 30-min intervals between continuous vibration (ON) and no vibration (OFF) over a 6–8 hr session. Movement activity, heart rate, respiratory rate, axillary temperature and blood-oxygen saturation were calculated separately for ON and OFF.

**Results:**

There was a 35% reduction in movement activity with SVS (p<0.001), with significantly fewer movement periods >30 sec duration for ON than OFF (p = 0.003). Incidents of tachypneic breaths and tachycardic heart beats were each significantly reduced with SVS, whereas incidents of eupneic breaths and eucardic heart beats each significantly increased with SVS (p<0.03). Infants maintained body temperature and arterial-blood oxygen level independent of stimulation condition.

**Conclusions:**

SVS reduced hyperirritability and pathophysiological instabilities commonly observed in pharmacologically-managed opioid-exposed newborns. SVS may provide an effective complementary therapeutic intervention for improving autonomic function in newborns with Neonatal Abstinence Syndrome.

## Introduction

Drug withdrawal in newborn infants from drug exposure during pregnancy is a growing and costly public health problem due in large part to unprecedented maternal use and addiction to opioids [1–4]. Over the last decade there has been a 5-fold increase in the rate of hospital admissions nationwide for neonatal drug withdrawal, with recent estimates of ~6 per 1000 hospital births (1 infant every 25 min) [2–4]. In the United States, hospitalization costs to treat drug withdrawal in newborns more than doubled from $730 million to $1.5 billion between 2009 and 2012 due to increasingly more infants requiring prolonged hospitalization post birth (on average 3 weeks) for pharmacological management of withdrawal [2, 3].

Infants exposed to drugs *in utero* develop physical tolerance and dependence through placental transfer [5]. NAS is not an addiction or substance use disorder of the infant, but refers to the physiological adaptation that occurs from prenatal drug exposure and to the physical disturbance when the drug is terminated at birth [6]. The abrupt cessation of drug transfer that occurs when the infant’s blood supply is severed from their mother’s at delivery results in dysregulation of central and autonomic function, which can be life threatening [5, 7]. Neonatal Abstinence Syndrome (NAS) refers to a variety of drug withdrawal signs and dysregulated behaviors, particularly prevalent in newborns with opioid exposure [8]. Clinically significant physiological signs of NAS include hyperirritability defined by increased movement (e.g., hypertonicity, jitteriness, and prolonged crying/wakefulness) [9], as well as seizures, irregular patterns in breathing and heart rate, and problems with thermoregulation (fever, sweating) and feeding (poor intakes, vomiting, diarrhea) [7–9]. Non-pharmacological strategies such as minimizing environmental stimuli, swaddling and positioning, and improving caloric intake are considered as the first-line treatment of infants with NAS [10–14], and there is some recent evidence that parental rooming-in models may reduce withdrawal symptoms, facilitate weaning, decrease length of stay and reduce pharmacotherapy requirement [15, 16]. However, many of these strategies may not be feasible or effectively carried out. Most infants with NAS require pharmacological treatment (e.g., morphine, methadone, clonidine) to manage physiological withdrawal [4, 10, 12], which may also independently contribute to long-term consequences on development [17–22]. Effects of medication and non-pharmacological interventions on autonomic, sensory, and motor activity remain largely unstudied [7, 8, 12, 14].

The purpose of this study was to assess the therapeutic potential of stochastic (random), vibrotactile stimulation (SVS) using a uniquely-constructed crib mattress as a complementary intervention for NAS in newborn infants exposed to opioids *in utero*. There is evidence that low-level, stochastic stimulation can promote stability in destabilized biological systems [23, 24], including improved cardiac and respiratory function in extremely vulnerable premature infants [25, 26]. In animal models, artificial tactile stimulation reorganizes brain structures and improves behaviors and physiological function [27–29]. We hypothesized that SVS would transform destabilized central and autonomic function and reduce symptoms of NAS, indicated by a reduction in hyperirritability (indexed by movement activity) and pathophysiological instabilities of heartbeat, respiration, and temperature.

## Methods

### Human subjects

A prospective study was performed on 26 full term infants (GA>37wks) at the University of Massachusetts Memorial Medical Center Neonatal Intensive Care Unit and Newborn Nursery, Worcester MA. Infants with documented fetal opioid exposure (confirmed by positive meconium toxicology, except for Subject #13 who was identified by maternal self-report for use of Vicodin during pregnancy), with and without polydrug exposure (Table 1), and hospitalized since birth with moderate to severe withdrawal defined by need for pharmacological treatment for NAS (as per standard of care NAS treatment protocol) were identified to study investigators by the attending physician (Table 1). Infants were not considered for study if they did not meet these criteria or had congenital abnormality, anatomic brain anomaly, hydrocephalus or intraventricular hemorrhage >grade 2, seizure disorder not related to drug withdrawal, clinically significant cardiac shunt, anemia (hemoglobin<8g/dL), and/or infection at time of the study session.

**Table 1:** Characteristics of the study population.

	Value	SD
**Infant**		
Gestational age, wk	39.5	1.2
Post Conceptual Age (*day of study*, wk)	41.5	1.7
Birth weight, g	3010	405
Weight *day of study*, g	3203	586
Birth head circumference, cm	32.6	1.4
Head circumference *day of study*, cm	34	1.5
APGARs 1min/5min	7.2/8.7	2.4/0.8
Male gender, n	16	
Morphine dose *day of study*, mg/kg	0.06	0.04
Number of days on morphine *day of study*, n	13	10
Also on phenobarbital *day of study*, n	8^†^	
Feed type day of study: breastmilk/formula/both, n	1/17/8	
Finnegan score max/mean day before study*	9.2/6.8	2.9/2.4
Finnegan score max/mean day of study*	9.7/7.0	3.0/2.4
Finnegan score max/mean day after study*	8.8/6.4	2.8/2.2
Paired stimulation trials 1/2/3/4/5, n	3/9/7/3/4	
**Mother**		
Age at infant’s birth, yr	28.4	4.7
Weight day of infant delivery (n = 25), lb	173	30
Methadone/Buprenorphine only, n	9/2	
Methadone plus other drugs (illicit or prescription)/plus unknown, n	8/3	
Non-maintenance opioid/s plus other drugs (illicit or prescription), n	4	
Smoked during pregnancy, self-report, n	23	
Delivery mode: vaginal/cesarean-section, n	21/5	
Anesthesia delivery medication	23	
Premature rupture of membranes ≥ 1hr, n	21	

Data are presented (for n = 26) as mean ± SD unless indicated otherwise; n = number of subjects.

^†^An additional 4 subjects were administered phenobarbital subsequent to study day.

*No significant difference among Finnegan scores between days of study for max or for mean (separate repeated measures analysis of variance, p>0.10)

NAS severity was routinely assessed by the infant’s bedside nurse trained on a modified version of the Finnegan Scale [30] that was restructured as part of a separate quality-of-care improvement initiative to increase scoring-consistency among nurses and minimize infant disturbance. The form was reorganized to clarify *when* to score signs of withdrawal, provided detailed scoring guidelines, and retained the Finnegan summed score over ~4 hour intervals [30]. Hospital records, chart review and questionnaire were used to obtain demographic and medical history on the infant and biological mother, including toxicology report (Table 1). Written informed consent was obtained from the biological mother of each infant enrolled. The study was approved by the University of Massachusetts Medical School Institutional Review Board for Human Subjects.

### Study design

A single-session, within-subjects design was used to compare effects of SVS to no stimulation. Movement, heart rate, respiratory rate, axillary temperature and arterial-blood oxygen saturation were quantified by continuous physiological measurement with each infant serving as his or her own control.

#### Physiological measurements

Respiratory inductance plethysmography (Somnostar PT, Viasys Healthcare, Yorbalinda, CA) was used to record abdominal and thoracic respiratory movements. Electrocardiographic activity (ECG) was recorded using surface electrodes over the infant’s chest in a 3-lead configuration. A pulse oximeter (Masimo, Irvine, CA) on the infant's foot measured transcutaneous arterial-blood oxygen saturation (SaO_2_). The quality of the plethysmographic (QPleth) activity characterized in the pulse-oximeter signal was used to identify movement periods [31]. Skin temperature was recorded from the infant’s axilla (Physitemp TH-5, Clifton, NJ).

#### Environmental and behavioral measurements

A sound meter (ExTech Instruments, Nashua, NH) and light meter (AEMC, Industrial Process Measurements, Dover, NH) by the infant's head recorded sound intensity (dBA) and changes in light levels (lux), respectively. Investigator observations, nursing/caregiver interventions (e.g. feeding, pharmacological dosing, diapering) and experimental conditions were recorded as time-stamped comments synchronized with the signals. Time-stamped comments and video recordings were used to identify interventions and technical contamination.

#### Vibrotactile stimulation

The infant’s mattress was replaced with one of two specially-constructed mattresses (23”x12”x3”) that fit into the standard hospital crib (26”x14”x8”) and provided whole-body SVS (30-60Hz, 10–12μm RMS surface near-linear surface displacement; TheraSound, Bellingham, WA or Wyss Institute, Harvard University). Previous findings demonstrated that such mattress SVS improved cardiac and respiratory function without increased arousal or movement activity in premature infants [25, 26].

### Data acquisition

All physiological, behavioral and environmental signals and analog output to the mattress were recorded and displayed during the experiments and stored on hard disk for offline analysis (Embla N7000, Broomfield, CO). Respiratory signals were sampled at 50Hz, ECG at 2000Hz, QPleth at 100Hz, SaO_2_ at 10Hz, mattress at 200Hz, and temperature, light and sound each at 20Hz. Overt behavioral data recorded with a video camera (MicroCamera, Panasonic, Newark, NJ) within the infant's crib area were synchronized with the physiological, audiometry and light signals.

### General procedures

**Fig 1:**
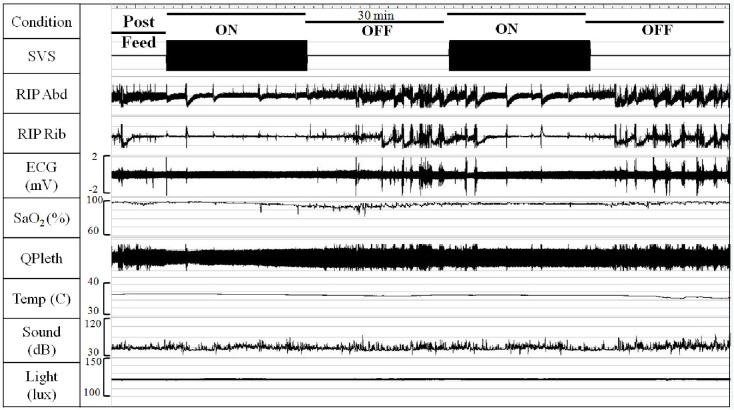
Example of experimental protocol and recorded signals (Subject 24). Signals are condensed. SVS = Stochastic Vibrotactile Stimulation; RIP = Respiratory Inductance Plethysmography; Abd = Abdomen; ECG = Electrocardiogram; SaO_2_ = Blood-oxygen saturation; QPleth = Quality of Plethysmography; Temp = Temperature. Note the increase in movement artifact in the physiological signals during OFF compared to ON.

Studies were conducted between ~8am and 6pm. To minimize infant disturbance, sensors were attached to the infant after nurse assessments and diaper change. Following feeds (formula or breastmilk; Table 1), the infant was placed supine in his/her crib for a 30-min observation period to ensure integrity of the recordings and allow the infant to resume sleep. Mattress stimulation was alternated in 30-min intervals between continuous vibration (ON) and no stimulation (OFF) throughout inter-feed periods (Subject 1 received 20-min and Subject 3 received 15-min intervals due to technical protocol-adjustment at onset of the study). The order of the ON-OFF cycles was randomized across subjects and counterbalanced between feeding periods within subjects. Fig 1 shows an illustration of a typical experimental protocol and recorded signals.

### Data processing and analysis

Signal analyses were performed using automated programs, described below. The number of paired ON/OFF trials varied among subjects (Table 1) due to factors beyond study control: e.g., inter-feed durations (infants were on-demand feed), continual touching by parent, or excessive irritability during 30-min post feed interval wherein infant did not settle and was placed by nurse in prone position, in alternative hospital-issued vacillating seat, or held by caregiver. Conditions that did not have a matched ON/OFF pairing were excluded. For subjects having multiple paired ON/OFF trials, a single mean was calculated for the ON conditions and for the OFF conditions so that subjects were equally weighted for each analysis.

#### Total condition time

For each ON and OFF condition, total condition time was calculated after excluding periods of nurse/caregiver interventions and contamination by technical sources.

#### Movement period (MP)

**Fig 2:**
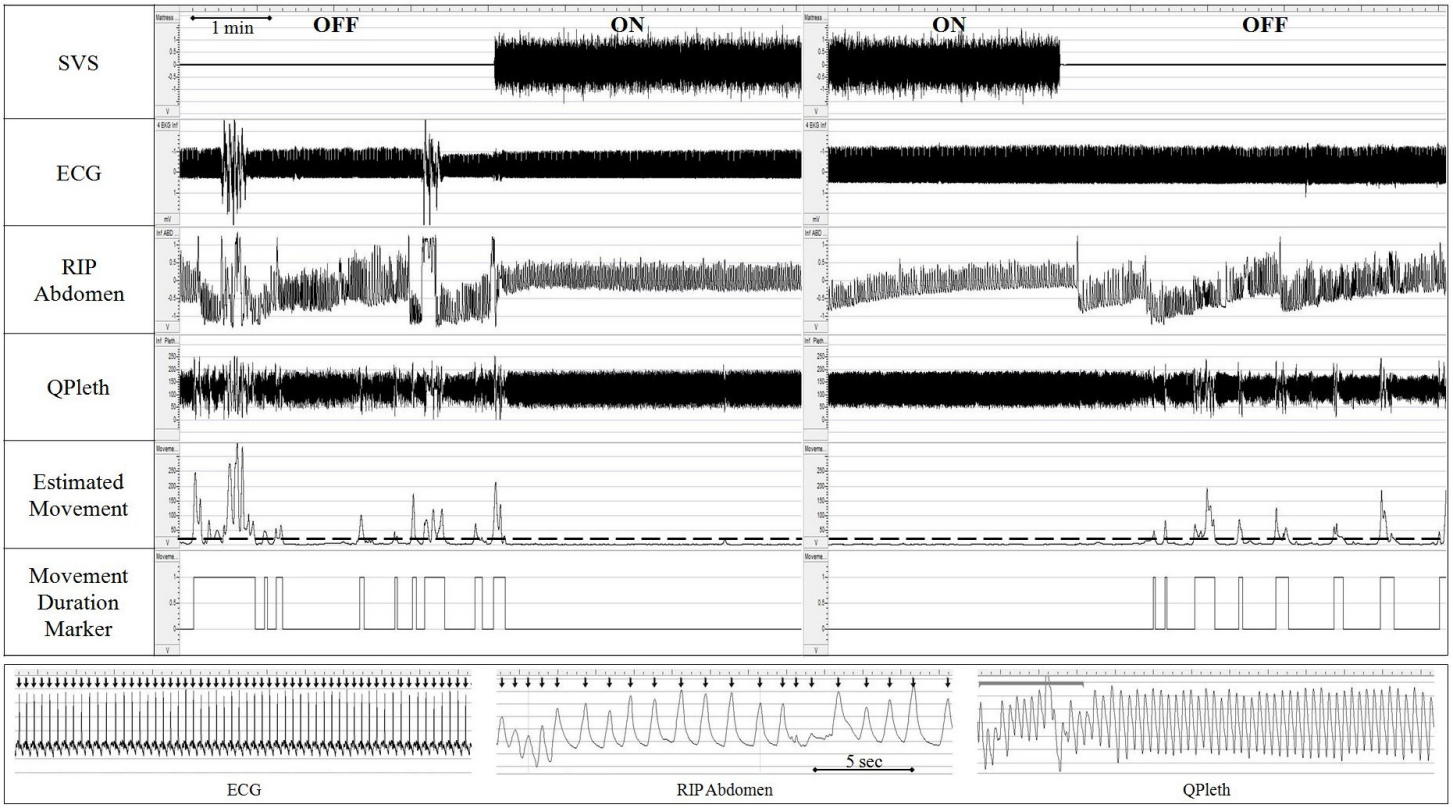
Physiological response to SVS. Upper panel: Improved cardio-respiratory stability and reduced movement with stimulation. Lower panel: Expanded view: 1) ECG: Arrows indicate heart rate calculated from cardiac R-waves; 2) RIP Abdomen: Respiratory inductance plethysmography; Arrows indicate respiratory rate determined from inspiratory peak; 3) QPleth: Gray line indicates distortion from movement activity.

For each condition, MP was defined by infant movement that generated distortion in the QPleth (Fig 2). A time-series signal estimating movement that captured this distortion was derived using a wavelet-based algorithm [31]. A binary marker indicated start of a MP when the time-series exceeded a threshold of 20, and indicated stop of MP when time-series fell below this threshold (Fig 2). Movements with inter-movement intervals ≤5 sec were merged into a single MP.

Movement period duration was expressed as a percent (MP%) of total condition time and quantified per unit of total condition time (in hr). Mean MP durations were quantified for short, medium, and long MP bursts: ≤5 sec (index of quick movement bursts common with myoclonic jerks, moro reflex, hypertonicity); >5and≤30 sec (brief arousal); and >30 sec (full arousal/behavioral wakefulness [32, 33]), respectively.

#### Valid non-movement condition time

For each subject, valid non-movement condition time was obtained after excluding periods of infant movement, interventions, and contamination (total condition time minus movement period). Because excessive movement tended to contaminate the morphology of the respiratory signal resulting in ambiguous signal detection, for QPleth distortions >5 sec the corresponding periods of respiratory, ECG, SaO_2_, temperature, light and sound signals were excluded from data analyses. Contiguous measurements of the non-contaminated portions of the physiological and environmental signals were analyzed for each condition and normalized per unit for valid non-movement condition time/hr.

#### Inter-breath intervals (IBI) and respiratory rate

Respiratory inductance plethysmography of the abdominal movements was used to generate a time series of IBIs (sec), determined from the peak of the inspiratory signal using automated peak-detection software (Fig 2; LabChart 7, ADI Instruments, Colorado Springs, CO). Mean respiratory rate was calculated from mean IBI rate for each condition. Mean incidence of IBIs was quantified across a range of respiratory rates observed in healthy newborns [34]: 1) Tachypnea (IBI ≥0.3 and ≤1.0 sec; respiratory rate ≥60 and ≤200 breaths/min); 2) Eupnea (IBI >1.0 and ≤2.0 sec; respiratory rate ≥30 and <60 breaths/min); 3) Bradypnea (IBI >2.0 and ≤10.0 sec; respiratory rate ≥6 and <30 breaths/min); and 4) Apnea (IBI >10 sec; respiratory rate <6 breaths/min), i.e., non-obstructive, central pauses in breathing associated with lack of effort in both the abdominal and rib plethysmographic activity. Variance of the IBI distribution, a measure of breathing stability, was also determined.

#### Cardiac intervals

R-R intervals were calculated using an automated peak detection program for each condition (Fig 2; Matlab, MathWorks, Natick, MA). For each subject, mean and variance of heart rate were calculated for each condition. Mean incidence was calculated also across a range of heart rates observed in healthy newborns [35, 36]: 1) Tachycardia (≥150and<300 bpm); 2) Eucardia (≥100and<150 bpm); and 3) Bradycardia (<100 bpm).

#### Oxygen saturation levels (SaO_2_)

Mean and variance of SaO_2_ (%) were calculated for each condition.

#### Axillary skin temperature

Mean and variance of infant skin temperature (C°) were calculated for each condition, within the range between 34.0 and 38.0C.

#### Ambient sound and light levels

Mean ambient sound level (dBA) and light level (lux) were calculated for each signal for each condition.

### Statistical analysis

Statistical calculations were performed using commercially available software (SPSS version 21, Chicago, IL; and SAS version 9.3, SAS Institute, Inc, Cary, NC). To characterize the distribution of the movement burst durations, histograms were analyzed for each condition for each subject (Maximum Likelihood Estimator; Matlab, MathWorks, Natick, MA). Sigma, an index of skewness was obtained from the histograms of the MP durations for each condition. Unadjusted comparisons of outcomes between SVS OFF and SVS ON were performed with pairwise t-tests. Separate repeated measures analysis of variance were used to compare mean and max Finnegan scores the day preceding, the day of, and the day after the study session. Finnegan scores relative to the 30min ON, 30min OFF intervals were not assessed because nurses’ Finnegan assessments are routinely conducted over a 4 hour period and would not reflect effect of SVS condition. Linear mixed model regression analyses were used to test for independent effects of Stimulation Condition (ON vs OFF), Mattress Type (Therasound vs Wyss), and infant and maternal variables on cardio-respiratory and movement activity. Because of the small sample size, we screened individual covariates for univariate relationships with the outcomes and those with a significant relationship with the outcome (p<0.1) were included in the parsimonious multivariate model as fixed effects. We did not have the power to test for interactions with SVS ON/OFF due to small sample size. For the adjusted SVS effect, a mixed effects regression model, adjusting for the within-infant correlation among the repeated measures, based on the individual significant fixed effects was subsequently fit separately for movement, heart rate and respiratory rate, modeling them as continuous outcomes. We did not have enough repeated measures within infant to obtain a stable estimate of the effect of time sequence separately. All values are expressed as means and 95% confidence interval [CI]. P values <0.05 were considered statistically significant.

## Results

### Clinical characteristics

Clinical characteristics and demographics are listed in Table 1. Infants studied were between post-conceptual age 38.3 and 44.7 wks. Twenty infants were exposed to methadone and two infants (Subjects 6 and 18) to buprenorphine for maternal maintenance treatment during pregnancy; of these, 11 infants were exposed only to a maintenance opioid and 11 infants had additional drug exposures. Four infants (Subjects 5, 9, 11, and 13) were exposed to non-maintenance opioids with polydrug exposure. On the day of the study, all infants were being treated with morphine for withdrawal, administered orally every 3 or 4 hours in accordance with standard of care at the time of the study; eight infants were also being treated for withdrawal with phenobarbital (Table 1). Morphine was administered primarily during nurse assessments preceding feeds (n = 19); four infants (Subjects 7, 11, 24, and 25) received morphine at the end of the final OFF condition, two infants (Subjects 12 and 18) received a morphine dose during an ON and OFF condition, and one subject (Subject 10) received morphine during an ON condition. Phenobarbital was not administered during the study period. Mean and max Finnegan scores [30] were not significantly different between the day preceding, the day of, or the day following the study session (Table 1).

### Condition and movement periods

**Fig 3:**
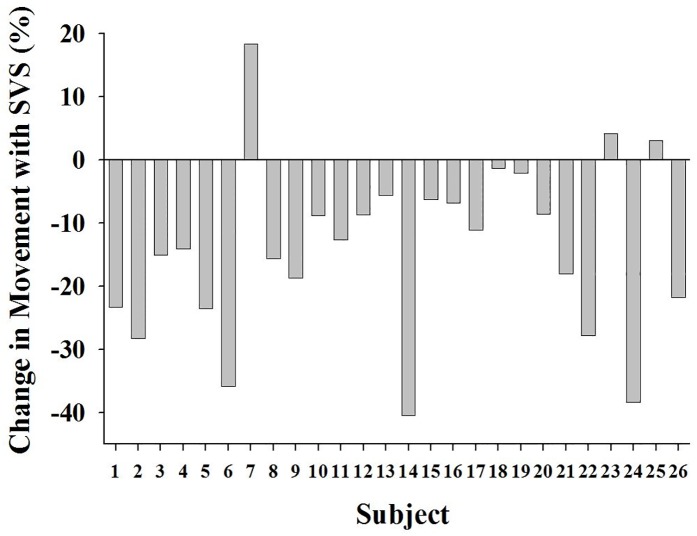
Change in movement with SVS among subjects. SVS reduced movement duration in 23 of the 26 subjects.

There was no difference in total condition time between OFF (mean = 28.7min, CI: 27.2–30.1) and ON (mean = 28.8min, CI: 27.4–30.2; p = 0.561); duration of caregiver intervention was not different between conditions (OFF mean = 58.1sec, CI: 25.8–90.4; ON mean = 54.5sec, CI: 12.1–97.0; p = 0.874). Valid non-movement condition time was significantly reduced for OFF (mean = 17.5 min, CI: 15.8–19.2) compared to ON (mean = 21.6min, CI: 20.0–23.2; p<0.001) due to a 35% relative reduction in MP% (14% absolute reduction) with stimulation (Table 2). A reduction in MP% with stimulation was observed in nearly all subjects (Fig 3).

**Table 2:** Effects of SVS on movement and cardio-respiratory activity.

Activity	SVS OFF	SVS ON	P Value
	Mean (CI)	Mean (CI)	
**Movement Period per VCT/hr**			
Mean Absolute Movement (%)	40 (36–45)	26 (23–30)	<0.001
Movement Periods < 5 sec (n)	27 (22–31)	26 (21–31)	0.692
Movement Periods 5–30 sec (n)	28 (23–33)	24 (20–27)	0.013
Movement Periods >30 sec (n)	13 (11–15)	9 (7–12)	0.003
**Respiratory Rate per VNMT/hr**			
Mean Respiratory Rate (bpm)	74 (68–80)	68 (61–75)	0.023
Interbreath Interval Variance (sec^2^)	0.13 (0.09–0.17)	0.16 (0.11–0.21)	0.303
Tachypneic Breaths (n)	2669 (2076–3262)	2184 (1572–2796)	0.024
Eupneic Breaths (n)	1184 (950–1418)	1392 (1150–1635)	0.028
Bradypneic Breaths (n)	64 (26–103)	72 (23–122)	0.559
**Heart Rate per VNMT/hr**			
Mean Heart Rate (bpm)	137 (133–141)	134 (130–138)	0.016
Heart Rate Variance ((bpm)^2^)	139 (107–171)	111 (81–142)	0.001
Tachycardiac beats (n)	1584 (965–2203)	1005 (522–1487)	0.009
Eucardiac beats (n)	6463 (6071–6855)	6920 (6604–7235)	0.011
Bradycardic beats (n)	19 (7–30)	25 (-2–52)	0.625

SVS = Stochastic vibrotactile stimulation; VCT = Valid condition time; VNMT = valid non-movement condition time.

Sigma, an index of histogram skew, revealed movement durations were spread over a wider range for OFF (mean = 1.7, CI: 1.6–1.8) than ON (mean = 1.5; CI: 1.4–1.6; p = 0.002). As provided in Table 2, there were significantly fewer incidents of prolonged MPs (>5and<30sec, and ≥30sec duration) for ON compared to OFF, whereas brief movement periods (≤5 sec duration) were not affected by stimulation condition.

### Physiological measurements

#### Respiratory

Fig 2 illustrates an example of improved stability of breathing with SVS in one infant (Subject 1). Among all infants, the mean incidence of eupneic breaths (≥30and<60 breaths/min) was significantly greater for ON than OFF, whereas the incidence of tachypnic breaths (≥60and≤200 breaths/min) was reduced with SVS (Table 2). The incidence of bradypnea was not affected by stimulation condition, and only 4 apneas (IBI>10 sec) total were observed among 3 infants. Group mean respiratory rate, while still above normal newborn range for both conditions [34], was on average 6 breaths/min lower with SVS (Table 2). Mean variance of IBI was not affected by stimulation condition (p = 0.303).

#### Cardiac and blood-oxygenation saturation

Table 2 provides the effects of SVS on heart rate. In summary, there was a large decrease (relative 36.6%) in the incidence of tachycardic heart beats and a significant increase in the incidence of eupneic heart beats with SVS, which likely contributed to the small but statistically significant reduction in heart rate (<3 bpm). The incidence of bradycardic beats was not affected by stimulation. Heart rate variance was also significantly reduced with SVS. There was no effect of stimulation on SaO_2_ (p = 0.544), which remained on average at 99% with negligible incidents of desaturation <85%.

#### Skin temperature

Mean axillary temperature was similar between both conditions (mean = 36.8°C; p = 0.581), with little variability observed throughout each condition (mean = 0.02; p = 0.116).

#### Environmental measurements

Ambient sound and light levels did not differ between conditions: mean sound levels were on average 52.2 dBA (p = 0.102) and mean light levels were on average 97.1 lux (p = 0.632).

### Exploratory models

Table 3 provides estimates of the effect of condition adjusted for maternal and infant variables on cardio-respiratory and movement response. Despite the small sample size, estimates of effects revealed that for ON there was a significant reduction in movement (14.6%), heart rate (2.6 beats/min) and respiratory rate (5.6 breaths/min) compared to OFF. Gender also played an important role, with females presenting with significantly lower movement (4.8%) and heart rate (11.4 beats/min) than males. Independent of condition, movement increased 2.9% per score on the Finnegan withdrawal scale, and respiratory rate increased at a rate of 2.21 breaths per score on the severity scale. Heart rate in infants with poly-drug exposure was significantly higher (6.6 beats/min) compared to infants with opioid-only exposure.

**Table 3:** Estimates of fixed effects for movement, heart rate, and respiratory rate.

Effect	Estimate (Effect Size)	Standard Error	DF	t Value	P Value
**Movement**	**% change**				
Stimulation ON (vs OFF)	-14.63	2.75	19	-5.32	<0.0001
Gender Female (vs Male)	-4.76	2.26	19	-2.11	0.048
Gestation Age (day)	-1.70	1.01	19	-1.69	0.107
Mattress Therasound (vs Wyss)	-6.11	2.56	19	-2.38	0.028
Withdrawal Severity (Mean Finnegan Score)	2.90	0.59	19	4.94	<0.0001
Maternal Weight (lb)	-0.12	0.04	19	-3.2	0.005
**Heart Rate**	**Beats/Minute change**				
Stimulation ON (vs OFF)	-2.62	1.70	18	-2.24	0.038
Gender Female (vs Male)	-11.35	2.34	18	-4.85	0.0001
Birth Head Circumference (cm)	-4.54	1.07	18	-4.24	0.0005
Study Head Circumference (cm)	6.64	1.05	18	6.31	<0.0001
Opioid + Poly Exposure (vs Opioid only)	6.60	2.26	18	-2.92	0.009
**Respiratory Rate**	**Breaths/Minute change**				
Stimulation ON (vs OFF)	-5.61	2.32	22	2.42	0.025
Gender Female (vs Male)	-8.97	5.93	22	-1.51	0.145
Withdrawal Severity (Mean Finnegan Score)	2.21	1.27	22	1.73	0.097
Maternal Age at Delivery	-0.40	0.62	22	-0.65	0.524

## Discussion

The important and novel finding of this study is that SVS reduced prolonged movement activity and improved cardiac and respiratory function in opioid-exposed newborns diagnosed with NAS. Results support our hypothesis that low-level, SVS impinges upon destabilized neural circuits to reduce hyperirritability and pathophysiological instabilities in drug-withdrawing newborns. Although little is known about mechanisms responsible for the effects, our findings suggest SVS may help regulate autonomic function by stimulating pressure receptors, specifically slowly-adapting pulmonary stretch receptors, to increase vagal tone (e.g., reduced tachypnea and tachycardia). Mechanosensory afferents may also impinge upon destabilized respiratory and cardiovascular brainstem oscillators to promote rhythmicity of the lungs (e.g, eupnea), heart (eucardia) and brain, crucial for healthy development [37].

Incidents of brief movements (≤5 sec) were not different between conditions suggesting hyperactivity of the CNS (hypertonicity, jitteriness)[9] and possibly active and quiet sleep [33, 38] are not affected by SVS. However, prolonged movement periods were significantly reduced with SVS, indicating that SVS may reduce arousals and improve sleep duration and organization [5, 33, 39]. A limitation to this study is that full-polysomnography was not used to assess sleep states. Nonetheless, increased prolonged movement activity observed during OFF is consistent with studies by O’Brien and colleagues who found increased movement, sleep disruption/fragmentation, and wakefulness in NAS infants compared to healthy controls, which were also related to withdrawal severity [33, 40]. In healthy newborns sleep deprivation impairs respiratory, cardiac, and arousal control mechanisms increasing risk for sudden infant death syndrome [41, 42] and may have implications for neurodevelopmental disorders [43, 44]. In adults and animal models, sleep deprivation results in increased pain sensitivity [45], which may partly explain opioid-induced hyperaglesia and hypersensitivity to stimuli often observed in drug-exposed infants [46]. Stochastic resonance may affect somatosensory and vestibular systems by facilitating more accurate detection of sensory inputs.

There were no adverse effects associated with SVS in the current study, though more studies are required to establish the safety and efficacy of SVS. Nurses and parents reported anecdotally that infants seemed “less irritable”, “calmer” and “slept better” during periods of SVS. We found virtually no thermoregulation or blood-oxygen desaturation issues throughout the study sessions, with infants maintaining their body temperatures and SaO_2_ levels independent of stimulation. Changes in sound or light environment were consistent throughout the conditions and likely did not contribute to the observed effects.

There is a great need for complementary, non-pharmacological therapeutic interventions for treating withdrawal in drug-exposed newborns. While the onset, duration and severity of symptoms are highly variable and unpredictable, withdrawal symptoms tend to be relatively similar regardless of drugs of exposure [9, 10, 47–49]. Current strategies for alleviating symptoms of withdrawal include non-pharmacological interventions such as swaddling, holding, skin-to-skin care, low-level environmental stimuli, rooming-in and non-nutritive sucking, but such strategies have not been well-studied [12, 15, 50]. Notably, NAS treatment is often dependent on pharmacotherapy, such as morphine, an opioid antagonist [4]. Evidence in animal models of drug exposure and in premature infants exposed to opioids post-partum for pain control suggest morphine may have long-term clinical consequences, including changes in behavior, brain function and higher-order neurocognitive processing (e.g., impulse control, attention, working memory) [17–19]. This is in addition to the adverse neurodevelopmental risks from *in utero* drug exposure [51–54]. Moreover, morphine does not fully reduce symptoms of withdrawal and often requires slow tapering. Infants in this study were all being treated with morphine to help manage withdrawal symptoms, yet continued to present with dysregulated behaviors during the study day despite pharmacological management. We found acute intervals of SVS significantly improved autonomic function. Because our intervention period was set at 30 min intervals, we could not assess the direct effect of SVS on the Finnegan [30] withdrawal scores since these assessments were performed over the procedural 3–4 hour period. Additional studies with longer stimulation intervals concurrent with Finnegan assessments are needed to determine if there are optimal SVS durations and phases of withdrawal in which SVS may be most effective, for example to reduce dose and duration of pharmacological capture, subsequent wean, and ultimately total pharmacological requirement.

This study demonstrated that brief intervals of whole-body SVS reduced cardiac and respiratory instabilities commonly observed in pharmacologically-managed opioid-exposed newborns. We took a conservative approach and excluded from analyses cardio-respiratory signals during periods of movement >5 sec, which likely also underestimated the effect of SVS on pathophysiological events (tachycardia and tachypnea) as more movement periods occurred in the OFF condition. Limitations are that this was a single session study in a small group of infants with various drugs of exposure, who presented with mild to severe withdrawal at the time of the study session and were studied at varying post-conceptual ages. Nonetheless, we found a very compelling effect of SVS. Exploratory analysis in this small group suggests that gender, withdrawal severity, and polydrug exposure may have independent effects on cardio-respiratory function and movement. Additional studies are needed to examine variables that may optimize the effect, including timing and duration of SVS. At the time of publication, these mattresses were not commercially available. The mattress prototype is designed for compatibility with standard neonatal equipment (e.g., fitting in bassinet polycarbonate baskets and isolettes/incubators) and easy to implement in the hospital setting. Longitudinal studies in more infants are warranted to determine whether a regimen of SVS therapy will reduce withdrawal symptoms sufficiently to allow lower doses and shorter durations of medication, maintain improved autonomic stability over time, reduce hospitalization and have significant implications on neurodevelopment for improved outcomes in this vulnerable population.

## Conclusions

SVS reduced hyperirritability and pathophysiological instabilities commonly observed in pharmacologically-managed opioid-exposed newborns. Findings suggest SVS may provide a safe, complementary non-pharmacological intervention for reducing undue movement activity, improving cardiac and respiratory function, and reducing symptoms of withdrawal in opioid-exposed newborns. The therapeutic potential of SVS for enhancing timely alleviation of opioid withdrawal requires further study in larger sample size to define optimal regimens and determine safety and efficacy. SVS could potentially serve as an effective, non-invasive complementary therapeutic intervention for improving autonomic function in newborns with NAS.
